# Complex roles of cAMP–PKA–CREB signaling in cancer

**DOI:** 10.1186/s40164-020-00191-1

**Published:** 2020-11-24

**Authors:** Hongying Zhang, Qingbin Kong, Jiao Wang, Yangfu Jiang, Hui Hua

**Affiliations:** 1 Laboratory of Oncogene, National Clinical Research Center for Geriatrics, West China Hospital, Sichuan University, Chengdu, China; 2 School of Basic Medicine, Chengdu University of Traditional Chinese Medicine, Chengdu, China; 3 Laboratory of Stem Cell Biology, West China Hospital, Sichuan University, Chengdu, 610041 China

**Keywords:** cAMP, PKA, CREB, Cancer

## Abstract

Cyclic adenosine monophosphate (cAMP) is the first discovered second messenger, which plays pivotal roles in cell signaling, and regulates many physiological and pathological processes. cAMP can regulate the transcription of various target genes, mainly through protein kinase A (PKA) and its downstream effectors such as cAMP-responsive element binding protein (CREB). In addition, PKA can phosphorylate many kinases such as Raf, GSK3 and FAK. Aberrant cAMP–PKA signaling is involved in various types of human tumors. Especially, cAMP signaling may have both tumor-suppressive and tumor-promoting roles depending on the tumor types and context. cAMP–PKA signaling can regulate cancer cell growth, migration, invasion and metabolism. This review highlights the important roles of cAMP–PKA–CREB signaling in tumorigenesis. The potential strategies to target this pathway for cancer therapy are also discussed.

## Background

Cell growth is tightly regulated by a variety of signal transduction pathways. Abnormal activation or inhibition of signal transduction pathways drives tumorigenesis. Posttranslational protein modifications, such as phosphorylation, ubiquitination, methylation and acetylation, are one of the important mechanisms that regulate cell signaling. Protein phosphorylation and dephosphorylation are regulated by various protein kinases and phosphatases, respectively. Abnormal or uncontrolled activation of protein kinases or phosphatases is very common in tumors, poising them as important targets for molecular targeted cancer therapeutics. As an inhibitor of the oncogenic kinase BCR–ABL, imatinib is the first kinase-targeted anticancer drug that has been successfully applied in the treatment of chronic myeloid leukemia [1]. Later on, inhibitors of protein kianses such as EGFR, ErbB2, MAPK, VEGFR and mTOR have been widely used in the treatment of a variety of common malignant tumors [2–5].

Cell signaling is often initiated by first messengers such as growth factors, hormones and ions, which trigger a series of signal transduction cascades via membrane receptors or intracellular receptors. This process involves multiple feedback mechanisms as well as a number of intracellular chemicals, known as the second messengers, such as cyclic adenosine monophosphate (cAMP), cGMP, calcium ions and so on [6]. In general, the production and distribution of these second messengers also need precise regulation. Abnormal production and distribution of these second messengers may contribute to carcinogenesis and tumor progression. The second messenger theory was first proposed by E. W. Sutherland in 1965. As a second messenger, cAMP is responsible for activation of the protein kinase A (PKA), exchange proteins directly activated by cAMP (Epac) and ion gated channel protein [7]. As one of the target proteins of PKA, cAMP response element binding protein (CREB) is an important transcription factor that regulates the expression of several genes including oncogenes c-Jun and cyclin D1 [8]. Notably, cAMP–PKA–CREB signaling has both tumor-suppressive and tumot-promoting effects on cancer, depending on the tumor types and context.

## The generation and degradation of cAMP

**Fig. 1:**
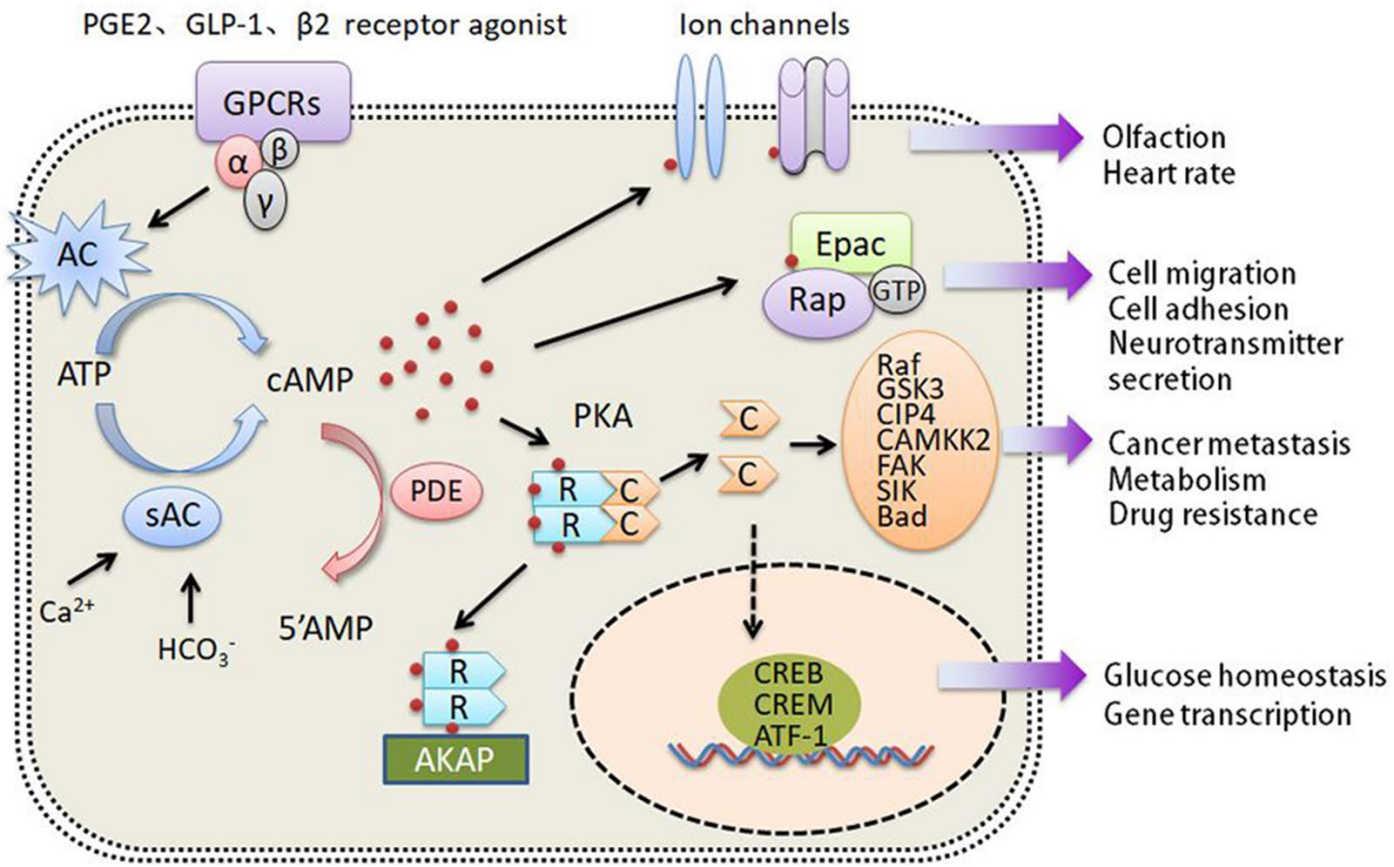
cAMP signaling pathway. The generation of cAMP is regulated in a G-protein dependent manner by AC or G-protein independent manner by sAC. The degradation of cAMP is regulated by PDEs. cAMP can regulate multiple signaling pathways including ion channes, Epac, and PKA. *R* the regulatory subunit of PKA, *C* the catalytic subunit of PKA

cAMP exists extensively in cells. Many hormones, neurotransmitters and other signaling molecules use it as intracellular second messenger. Therefore, cAMP can directly regulate various biological processes or behaviors of cells, including cell metabolism, ion channel activation, gene expression, cell growth, differention and apoptosis [9]. The generation of cAMP is regulated in a G-protein-dependent or G-protein-independent manner. After extracellular ligands, such as PGE2, GLP-1 and β2 receptor agonists, bind to G-protein coupled receptors (GPCRs), Gα subunits are separated from Gβ and Gγ subunits, and then activate adenylyl cyclases (ACs), leading to the conversion of ATP into cAMP (Fig. 1) [10–12]. In addition, bicarbonate (HCO_3_^−^) and calcium ions (Ca^2+^) induce cAMP synthesis by activating the soluble adenylyl cyclase (sAC) independent of G-proteins [13, 14]. In contrast, phosphodiesterases (PDEs) are responsible for the degradation of cAMP. So far, at least 22 PDEs have been identified [15]. The concentration of intracellular cAMP depends on the relative balance between adenylyl cyclases and phosphodiesterases.

## The molecular targets and roles of cAMP in cancer cell growth, migration and metabolism

As shown in Fig. 1, the major targets of cAMP include PKA, Epac1 and Epac2, and nucleotide-gated ion channels. The cAMP binding domain of Epac can bind a cAMP molecule, resulting in conformational changes of the protein thereby exposing the active site in catalytic domain. As a guanosine exchange factor (GEF), Epac can promote Ras family proteins Rap1 and Rap2 to release GDP and bind GTP, leading to the activation of Rap1/2 [16]. Epac regulates multiple cell processes mainly through Rap-GEF dependent and Rap-GEF independent pathways, including migration and focal adhesion formation, morphogenesis, cardiac output, neurotransmitter release, glucose homeostasis, exocytosis, cell proliferation, cell differentiation and cell survival [16]. cAMP can also bind to some nucleotide-gated ion channels and regulate their functions, such as cyclic nucleotide gated ion channels (CNG) and hyperpolarization-activated cyclic nucleotide-gated channel (HCN) [6]. These channels are non-selective cation channels that can conduct calcium, sodium and potassium ions and change the cell membrane potential. Below, we shall focus on PKA as a classical target of cAMP.

PKA is a tetramer enzyme consisting of two regulatory subunits (R) and two catalytic subunits (C) (Fig. 1). In mammals, there are four types of regulatory subunits, namely RIα, RIβ, RIIα and RIIβ. According to the different regulatory subunits, PKA can be classified into PKA type I (RIα_2_C_2_, RIβ_2_C_2_) and PKA type II (RIIα_2_C_2_, RIIβ_2_C_2_) [17]. The binding to two regulatory subunits of the inactive PKA tetramer by cAMP results in the release and activation of the catalytic subunits, phosphorylation of the serine and threonine residues in substrate proteins [18]. RIα and RIIα are widely distributed in different cells, while RIβ mainly exists in the brain, testis and B lymphocytes, and RIIβ is expresses in the brain, fat, and some endocrine tissues [19]. The localization of the tetramer enzyme is also different. PKA type I are generally cytoplasmic, while PKA type II are specifically anchored in subcellular structures and compartments [20]. PKA anchored proteins (AKAPs) can bind to cytoskeleton proteins or organelles and to PKA regulatory subunits, thereby playing an important role in the distribution of PKA in cells and enabling PKA to park and concentrate key targets [21, 22]. PKA type II is generally expressed in normal non-proliferating tissues and cells in growth retardation phase. PKA type I expression can be temporarily induced by physiological stimulation in cell proliferating phase and stimulated by certain growth factors and receptors, such as EGFR, TNF-α, and ErbB-2. Moreover, PKA type I is overexpressed in a variety of primary tumors [23].

There are three types of PKA catalytic subunits, including Cα, Cβ and Cγ. Cα and Cβ are two major types and have multiple splice variants. This subtype diversity is an important mechanism for achieving the specificity of PKA signaling [24]. Once activated, PKA phosphorylates its substrates, such as CREB, Raf, Bad and GSK3 [25–27], and then regulates gene expression, cell survival and migration. Recent study indicates that PKA is an actomyosin contractility-regulated effector of cellular mechanotransduction and a regulator of mechanically guided cell migration [28]. PKA also phosphorylates CDC42 interacting protein 4 (CIP4), a coordinator of membrane deformation and actin polymerization, and promotes cancer cell invasion and metastasis [29]. Actually, the promotion of cancer cell invasion and metastasis by PKA may involve multiple PKA substrates. The focal adhesion kinase is another PKA target that mediates the promotion of cancer metastasis by cAMP [30].

In addition, PKA can phosphorylate and then inactivate the calmodulin-dependent protein kinase kinase-2 (CAMKK2) [31]. CaMKK2 plays important roles in energy homeostasis, insulin signaling and whole-body metabolism [32, 33]. Inhibition of CAMKK2 protects against prostate cancer, hepatocellular carcinoma (HCC) and metabolic disorders induced by a high-fat diet [34]. Moreover, PKA can reprogram lipid metabolism by inhibiting salt-inducible kinases, and then promote pancreatic tumorigenesis [35]. Meanwhile, PKA signaling cooperates with the epigenetic regulators JMJD3 and SIRT1 to activate β-oxidation-promoting genes [36]. There may be more and more metabolism-related targets of PKA to be uncovered. Of note, activated PKA can feedback inhibit adenylyl cyclase (AC5 and AC6) and activate phosphodiesterase (PDE3 and PDE4) thereby lowering the levels of cAMP and maintaing cAMP homeostasis [12, 37].

**Fig. 2:**
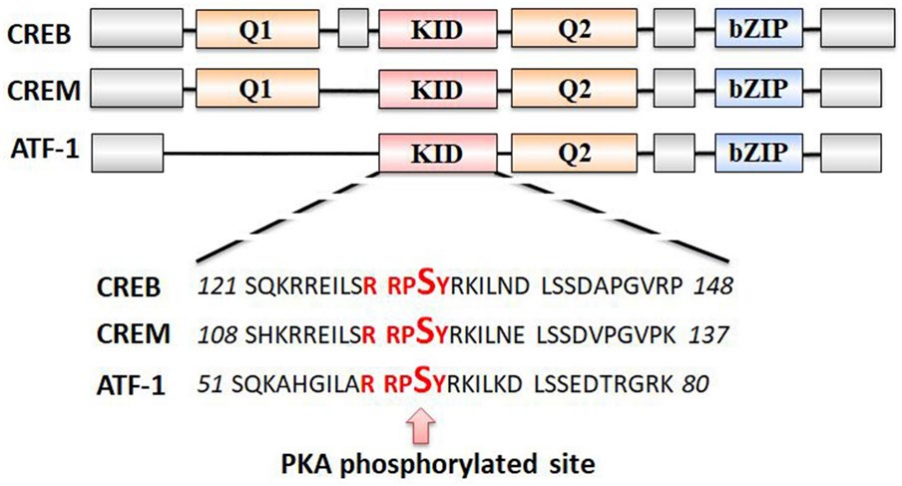
The domains of CREB family proteins. The CREB family proteins contain a kinase inducible domain (KID), two glutamate domains (Q1 and Q2), and a basic leucine zipper domain (bZIP)

The CREB family proteins are well-characterized PKA substrates. This family is a group of the basic leucine zipper (bZIP) superfamily transcription factors, including CREB, cAMP responsive element regulatory protein (CREM) and transcriptional activator 1 (ATF1). CREB, CREM and ATF1 can form homodimers or heterodimers [38–40]. Both CREB and ATF-1 proteins are ubiquitously expressed, while CREM is mostly expressed in neuroendocrine tissues [40]. The CREB family proteins contain a kinase inducible domain (KID), two glutamate domains (Q1 and Q2), and a basic leucine zipper domain (bZIP). KID is a 60-amino acid fragment located in the central region of CREB, CREM and ATF1, which contains the PKA phosphorylation site (RRPSY) (Fig. 2) [41].

**Fig. 3:**
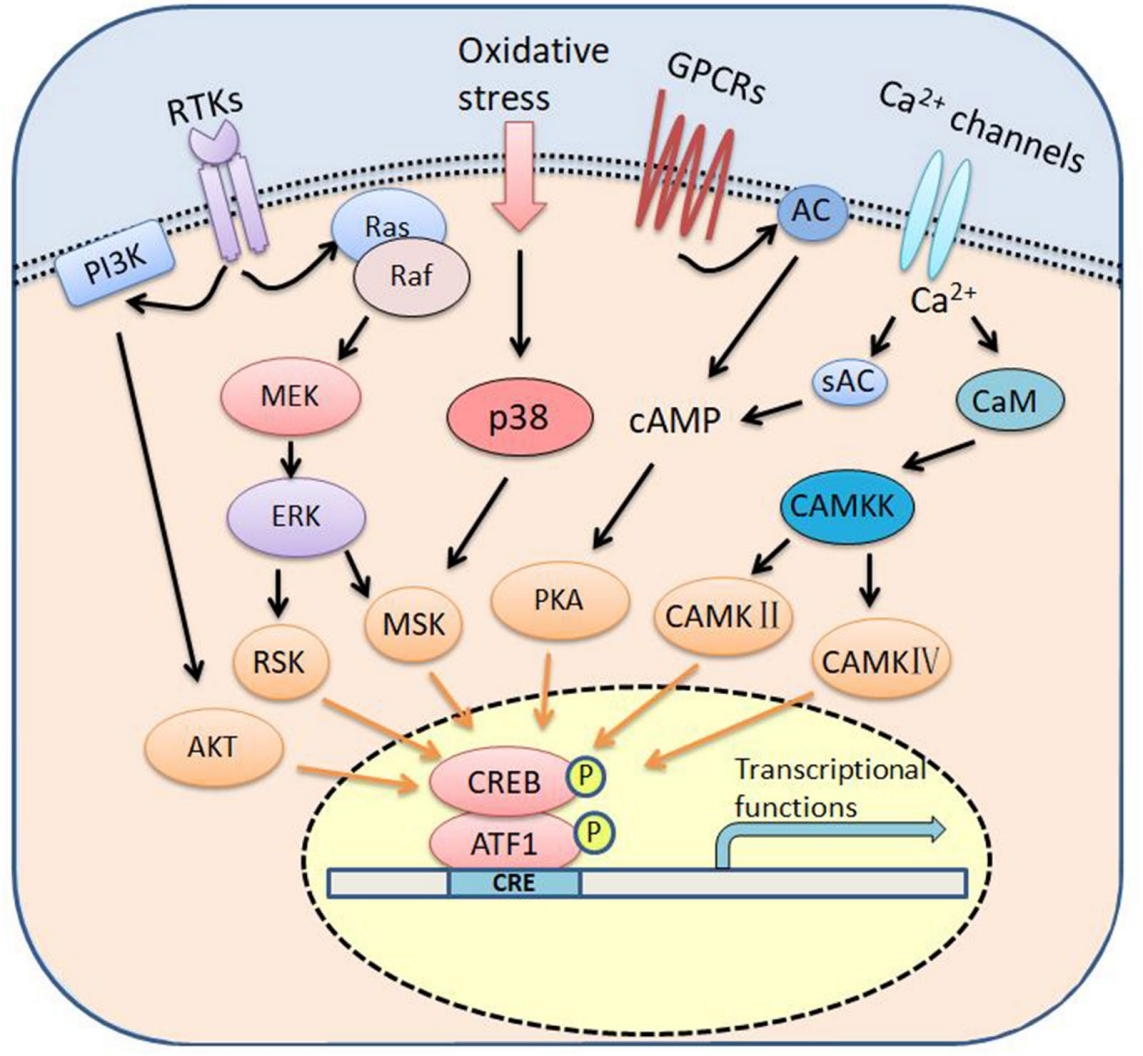
Cell signaling pathways for activating CREB/ATF1. CREB/ATF1 can be phosphorylated by multiple kinases including Akt, RSK, MSK, PKA, CAMKII and CAMKIV

Phosphorylation of CREB Ser^133^ residue and ATF1 Ser^63^ residue is critical for the transcriptional activity of CREB and ATF1. Activated ATF1 and CREB can form homodimer or heterodimer, bind to the cAMP response element (CRE) in the promoter region of target genes and initiate genes transcription, thereby regulating cell differentiation, proliferation, apoptosis, metabolism, glucose homeostasis, hematopoiesis, immune response as well as neuronal activities such as memory and learning (Fig. 3) [42–44]. In addition to the major PKA kinase, other kinases such as RSK, MSK, AKT, and CAMKII/IV also directly phosphorylate CREB and ATF1 [45–50].

## The roles of cAMP–PKA–CREB signaling pathway in tumors

cAMP–PKA–CREB signaling has paradoxical effects in tumors, acts as a tumor-suppressor or tumor-promoter in different tumor types (Table 1). The role of cAMP–PKA–CREB signaling pathway in liver cancer and other tumors is described below.

**Table 1:** The diverse functions of cAMP/PKA/CREB in human tumors

Cancer type	cAMP/PKA/CREB functions	References
HCC	DNAJB1–PRKACA gene fusion in FL-HCC patients promote tumor progression	[54, 55]
	Overexpression of PRKACA in BAP1-mutated HCC promotes tumor progression	[56]
	HBVx promotes liver carcinogenesis through CREB-miR-3188, CREB-YAP and Erk-CREB pathways	[57–59]
	cAMP analogues and PDE inhibitors inhibits HepG2 cell growth by down-regulating cyclin A and up-regulating p21/p27/p53	[52, 61]
Brain tumor	cAMP inhibits glioblastoma cell growth by up-regulating p21/p27 and PKA/ Epac1-Rap1 signaling	[63–65]
	PKA-Dock180 signaling promotes the development and invasion of glioblastoma	[74, 75]
	cAMP–PKA inhibits medulloblastoma by suppressing Hedgehog signaling	[66–68]
	CREB promotes glioma progression through down-regulating PTEN	[72, 76]
Lung cancer	cAMP can down-regulate SIRT6 expression and inhibit NSCLC cells apoptosis	[78, 79]
	PKA promotes hypoxia-induced epithelial-mesenchymal transformation, cell migration and invasion of lung cancer cells	[84]
	PKA induces PP2A phosphorylation and AP1, thereby increases radiotherapy-induced lung cancer cell apoptosis	[86, 87]
Prostate cancer	PKA can up-regulate AR signaling and the neuroendocrine differentiation of prostate cancer, leading to androgen-independence, resistance to androgen deprivation therapy and cancer progression	[81, 88–93]
Epithelial ovarian cancer	PKA promotes extracellular matrix degradation and reduces the intensity of tight junction in epithelial ovarian cancer cells by phosphorylating claudin-3, leading to tumor invasion and metastasis	[97–100]
Breast cancer	PKA promotes the growth and metastasis of triple negative breast cancer cells through GSK3-β-catenin pathway	[104]
	PKA induces ERα Ser^305^ phosphorylation, tamoxifen resistance and ER-positive breast cancer progression	[106, 107]
	PKA promotes trastuzumab resistance in Her-2 positive breast cancer	[108]
Leukemia and lymphoma	cAMP promotes TLR signaling and apoptosis of chronic lymphocytic leukemia (CLL) cells	[112, 113, 119–121]
	cAMP–PKA may reduce Bcl-2 and survivin expression and increase Bax expression in lymphoma cells, leading to cell apoptosis	[116–118]
	Overexpression of CREB in AML patients is associated with poor prognosis. Overexpression of CREB can promote AML cells proliferation by up-regulating cyclin A1 expression	[122, 123]

### Liver cancer

Whereas some studies suggest that increasing cAMP levels may inhibit HCC cells growth [51–53], it has been reported that PKA promotes HCC invasion and metastasis by phosphorylating multiple substrates such as CIP4 [29]. Fibrolamellar hepatocellular carcinoma (FL-HCC) is a primary liver cancer that occurs mainly in children and young adults. 80%-100% FL-HCC patients have DNAJB1–PRKACA gene fusion, resulting in the deletion of a 400 kb gene fragment on chromosome 19 and the production of a chimeric protein that retains PKA kinase activity [54]. DNAJB1–PRKACA knock-in mice can develop tumors characteristics of FL-HCC [55]. In addition, PRKACA is overexpressed in about 80% of BAP1 gene (encoding BRCA1-associated protein 1)-mutated HCC, which exhibit similar clinical manifestations and histological characteristics to DNAJB1–PRKACA fusion-related FL-HCC [56]. PKA pathway dysfunction plays an important role in the development and progression of these two kinds of HCC. Hepatitis B virus (HBV) infection is a major risk factor for the development of HCC. HBV X protein plays an important role in HBV-related HCC. Mechanistically, HBVx can promote liver carcinogenesis through CREB-miR-3188 and ZHX2-Notch signaling pathway [57], promote HCC cell growth by activating CREB-YAP axis [58], and promote the invasion and metastasis of HBV-related HCC by up-regulating FOXM1 expression through Erk-CREB pathway [59]. Collectively, these studies indicate that the PKA-CREB pathway may promote HCC progression. Indeed, analysis of rat HCC tumors and paired normal liver samples showed significant increase in CREB and CREB phosphorylation levels in HCC [60].

However, the roles of cAMP in HCC appear to be somewhat paradoxical. As we described above, increasing cAMP levels by PDE inhibitors may arrest HCC cells growth. Treatment of HepG2 cells with cAMP analogues significantly reduce the transcription and protein levels of cyclin A and induce cell cycle arrest [61]. In addition, PDE4 inhibitor rolipram and DC-TA-46 can up-regulate the expression of p21, p27 and p53 and down-regulate cyclin A expression, thereby inhibiting the proliferation and promoting apoptosis of HepG2 cells [52]. In contrast, vasoactive intestinal peptide could reduce cAMP concentration, CREB expression and Ser^133^ phosphorylation, and inhibit Bcl-xL expression, leading to Huh7 cell apoptosis [62]. The versatile roles of cAMP in HCC may be due to the multiplicity of its targets with diverse functions. Hence, whether cAMP promotes or inhibits HCC may be context-dependent. The homeostasis in cAMP levels may be critical for HCC progression.

### Brain cancer

The roles of the CREB-activating kinase PKA in brain tumors are also paradoxical. Several studies have shown that PKA plays a tumor-suppressive role in glioblastoma cell line A-172. Activation of PKA by increasing cAMP levels or supplying cAMP analogues (dcAMP and 8-Br-cAMP) can reduce the proliferation rate of A-172 cells, promote differentiation, and induce apoptosis [63]. Increasing the intracellular cAMP levels by rolipram, a PDE inhibitor, can up-regulate the expression of p21 and p27, and activate PKA and Epac1-Rap1 signaling, leading to A-172 cell growth arrest and apoptosis [64, 65]. cAMP pathway also plays anticancer role in medulloblastoma. Pituitary adenylyl cyclase inhibits the proliferation of medulloblastoma cells through PKA-Gli1 pathway [66], and neurociliary proteins inhibit the growth of medulloblastoma through PDE4D-PKA-Hedgehog pathway [67]. ARHGAP36 protein, a member of RhoGAP family, can bind to PKA catalytic subunit and inhibit its activity, thereby activating Hedgehog pathway and promoting the growth of medulloblastoma [68]. The tumor-suppressive effects of cAMP and PKA may be mediated by Epac1, Rap1 and Gli1, rather than CREB.

Phosphorylation of CREB can be directly inhibited by PTEN, an anticancer protein phosphatase that is frequently mutated or inactivated in many cancers, including the most aggressive types of brain cancer, glioblastoma multiforme and astrocytoma [69, 70]. PTEN deficiency can enhance CREB activity and induce the expression of PAX7, thereby promoting the conversion of human neural stem cells to glioblastoma stem-like cells [71]. In addition, CREB can reciprocally down-regulate PTEN. Tan et al. reported that CREB was highly expressed in clinical samples and cell lines of glioma, and CREB could inhibit PTEN expression through miR-23, thus promoting the development of glioma [72]. Moreover, EGFR can activate CREB through MAPK-RSK2 pathway, and then promote glioma cells growth and invasion [73].

Epidermal growth factor receptor (EGFR), EGFRvIII mutant and platelet derived growth factor receptor (PDGFR) can promote the development and invasion of glioblastoma through the PKA-Dock180 signaling pathway [74, 75]. Also, miR-33a enhances cAMP–PKA signaling by inhibiting PDE8A expression, thus promoting the growth and self-renewal of initial glioma stem cells [76]. In human glioblastoma cell line MGR3, activation of PKA leads to a significant increase in GSTP1 phosphorylation and activity, which may lead to drug resistance and treatment failure [77].

### Lung cancer

In non-small cell lung cancer (NSCLC), there are significant up-regulation of CREB expression and phosphorylation in tumor tissues compared with adjacent normal tissues. Increased CREB expression is correlated with short survival period of patients [78]. cAMP could down-regulate SIRT6 expression and thus reduce the apoptosis of NSCLC cells induced by radiotherapy [79]. In the highly malignancy small cell lung cancer (SCLC), increased activity of CREB helps maintain its neuroendocrine characteristics and proliferation [80]. RGS17 is increased in 80% of lung cancer tissues compared with matched normal lung tissue and promotes cell proliferation through the cAMP–PKA–CREB pathway [81]. In addition, PKA-Smurf1-PIPKIγ signaling transduction promotes the progression of lung cancer in vivo [82]. cAMP–PKA–CREB pathway could regulate the hypoxia response in lung cancer cells [83]. PKA inhibitors H-89 and PKACA knockdown antagonize hypoxia-induced epithelial-mesenchymal transformation, cell migration, and invasion of lung cancer cells [84]. Moreover, oxidative stress plays an important role in the pathogenesis of lung diseases, including pulmonary fibrosis and lung cancer. Interactions among PKA, Erk1/2 and CREB mediate cell survival in oxidative stress [85].

Contradictory to the inhibition of radiotherapy-induced NSCLC cell apoptosis by cAMP-Sirt6 pathway [79], cAMP–PKA–CREB pathway seems to play an anticancer role in radiotherapy. BALB/c mice pretreatment with forskolin could inhibit ATM and NF-κB by PKA-induced PP2A phosphorylation, resulting in an increase in radiotherapy-induced apoptosis [86]. In lung cancer cell line H1299, Gs protein promotes Bak expression through PKA-CREB-AP1 pathway and increases apoptosis induced by radiotherapy [87]. These studies suggest that the cAMP–PKA–CREB pathway may also exert opposite effects under different circumstances of the same type of tumor.

### Prostate cancer

PKA subunit can be a biomarker to predict the response of prostate cancer to radiotherapy and chemotherapy. Analysis of 456 clinical prostate cancer specimens found that PKA RIα is highly expressed in 80 cases (17.5%), and PKA RIα overexpression is associated with poor efficacy of radiotherapy and short-term or long-term androgen deprivation therapy, distant metastasis and abnormal biochemical index [88]. In prostate cancer cell line PC3M cells, the overexpression of wild type PKA RIIβ or mutant type PKA RIα-P (functionally similar with PKA RIIβ) leads to growth inhibition and apoptosis in vitro, and inhibits tumor growth in vivo [89]. Androgen receptor (AR) signaling is critical for prostate carcinogenesis. Testosterone directly stimulates GPR56 and then activates the cAMP/PKA pathway, which promotes AR signaling [90]. PKA also phosphorylates the Thr^89^ residue in HSP90, leading to the release of AR from HSP90 and the binding of AR to HSP27, which transfers AR into the nucleus to transactivate its targets [91]. In addition, PKA signaling pathway is involved in the neuroendocrine differentiation of prostate cancer, an early marker for the development of androgen independence [92, 93]. Moreover, high expressions of osteocalcin and ostesialin in androgen independent prostate cancer cell line C4-2B are dependent on the cAMP–PKA signaling pathway [94]. PAK4 can be activated by cAMP–PKA to enhance CREB transcription activity independent of phosphorylation at Ser^133^ residue, and PAK4 knockdown in PC-3 and DU145 cells inhibit tumor formation in nude mice [95]. Moreover, RGS17 is overexpressed in prostate cancer samples and promotes cell proliferation through the cAMP–PKA–CREB pathway [81]. In addition, it has been reported that depression and behavioral stress can accelerate the progression of prostate cancer through PKA kinase [30, 96].

### Ovarian cancer

About 54% of epithelial-derived human ovarian tumors in tissue microarray have moderate or high levels of CREB expression, while no expression was observed in normal ovarian samples. Knockdown of CREB expression significantly reduces proliferation of ovarian cancer cells, but had no effect on apoptosis [97]. Epithelial ovarian cancer is one of the most deadly gynecologic malignant tumors. PKA RIα is highly expressed in epithelial ovarian cancer [98]. During metastatic spread of epithelial ovarian cancer cell line SKOV3, extracellular matrix invasion needs PKA activity and AKAPs anchor, and inhibition of PKA activity or PKA RI and RII anchor can block matrix invasion [99]. In addition, PKA reduces the intensity of tight junction in epithelial ovarian cancer cell line OVCA433 by phosphorylating claudin-3 [100]. Also, gonadotropin promotes the metastasis of epithelial ovarian cancer cells through PKA and PI3K pathways [101]. Whereas the above-mentioned studies suggest that PKA-CREB plays a tumor-promoting role in epithelial ovarian cancer, one study suggests that PKA can phosphorylate EZH2 at Thr^372^ residue, leading to mitochondrial dysfunction, the binding of EZH2 to STAT3 and then inhibiting STAT3 phosphorylation and epithelial ovarian cancer cell growth [102].

### Breast cancer

Overexpression of R subunit especially RI of PKA is associated with cell proliferation in normal breast, malignant transformation of breast epithelial cells, poor prognosis of breast cancer, and tolerance to anti-estrogen therapy. Recent study demonstrates that nuclear localization of activated PKA is correlated with breast cancer metastasis [103]. Integrin α9 maintains the stability of β-catenin through ILK/PKA/GSK3 signaling and, thereby promotes the growth and metastasis of triple negative breast cancer cells [104]. Moreover, successful anti-estrogen therapy is associated with reduced RI mRNA expression, and RI antisense oligonucleotides can reduce the growth rate of breast cancer cells [105]. In estrogen receptor positive breast cancer, PKA-induced ERα Ser^305^ phosphorylation and PAK1 are associated with tamoxifen resistance and breast cancer progression [106, 107]. In Her-2 positive breast cancer cells, PKA activation is associated with trastuzumab resistance [108]. In addition, cAMP–PKA–CREB pathway also plays an important role in the metabolic regulation of breast cancer. Serotonin promotes mitochondrial biosynthesis through the AC-PKA pathway in breast cancer cells [109]. Cytoplasmic G-protein coupled estrogen receptor promotes aerobic glycolysis through cAMP–PKA–CREB pathway [110]. Contrary to above studies, it is reported that IL-24 induces breast cancer cells apoptosis by activating TP53 and endoplasmic reticulum stress through PKA [111]. Hence, PKA may also have paradoxical roles in breast cancer depending on the context.

### Leukemia

The role of cAMP is quite different in diverse types of lymphoma. PDE4 inhibitors block intracellular TLR signaling and promote apoptosis of chronic lymphocytic leukemia (CLL) cells through increasing cAMP concentration [112, 113]. Interestingly, PDE4 inhibitors induce apoptosis in B cell CLL but not in T cell CLL or normal circulating hematopoietic cells, probably due to that PDE4 inhibitors only augment glucocorticoid receptor and cAMP levels in B cell CLL [114, 115]. cAMP–PKA could promote apoptosis through mitochondria dependent pathways, reducing expression of anti-apoptotic proteins Bcl-2 and survivin, and increasing expression of pro-apoptotic protein Bax in lymphoma cells [116–118]. The chemokines CXCR4 and CXCL12 released from the microenvironment can bind to Gαi-conjugated GPCRs on CLL cells, reducing cAMP synthesis and increasing survival rate of CLL cells [119, 120]. PDE7B is overexpressed in CLL, and inhibitors of PDE7 (BRL-50481 and IR-202) and a dual PDE4/PDE7 inhibitor IR-284 increase apoptosis of CLL cells, which is attenuated by PKA inhibition [121].

CREB is overexpressed in the bone marrow of most leukemia cell lines and patients with acute myeloid leukemia (AML) and acute lymphoblastic leukemia (ALL) [122]. Previous studies also show that CREB is highly expressed in the majority of myeloid leukemia cells in AML patients and associated with poor prognosis [123]. The jmjd3/UTX inhibitor GSKJ4 can promote CREB degradation and inhibit AML cell growth [123]. Overexpression of CREB can promote AML cells proliferation by up-regulating cyclin A1 expression. In addition, the CREB transgenic mice shows myeloproliferative diseases but not leukemia, suggesting that CREB is involved in the leukemia phenotype during the leukemia germination, but is not sufficient to completely transform into leukemia [124]. In contrast, cAMP can protect acute promyelocytic leukemia cells from apoptosis induced by arsenic trioxide and anthracyclines [124]. In AML cell line IPC-81, cAMP can induce apoptosis through up-regulation of Bim by CREB and CDK [125].

### Other tumors

The mRNA and protein levels of PKA RIα and AKAP10 are significantly increased in colorectal cancer tissues, correlating with invasion depth, differentiation degree and short survival [126]. Type-I insulin-like growth factor receptor (IGF-IR) is tightly involved in tumorigenesis and drug resistance [127]. IGF-IR signaling induces ezrin phosphorylation at Thr^567^ residue and thereby promotes cAMP-dependent PKA activation and colorectal cancer cell survival [128]. Overexpression of PKA RIα and AKAP10 in several colorectal cancer cell lines is directly correlated to metastasis. In addition, the resistance of colorectal cancer cells to a selective MEK1/2 inhibitor selumetinib is induced by PKA activation [129], and the resistance of colorectal cancer cells to methotrexate can be induced by cAMP signaling [130]. PKA antagonists could inhibit the nuclear translocation of β-catenin and expression of c-myc and COX2 in APC mutant colorectal cancer, thereby inhibiting tumor development [131].

Immunohistochemical experiments found that normal melanocytes did not express PKA RIα proteins, but it is highly expressed in human melanoma samples and some melanoma cell lines. RII activation or RIα silence can inhibit proliferation and increase caspase 3-promoted apoptosis [132]. PKA can promote the migration of melanoma cells. Studies have found that hypoxia can induce the expression of scaffold protein AKAP12 in melanoma, and PKA-regulated phosphorylation during hypoxia is dependent on the presence of AKAP12. Inactivation of AKAP12 leading to the reduction of tumor growth, migration, and invasion in melanoma mouse models [133]. PKA pathway also plays an important role in the synthesis of melanin. Diethylstilbestrol can promote melanin production through cAMP–PKA mediated up-regulation of tyrosinase and MITF in mouse melanoma cell line B16 [134], and gingerol inhibits melanin production by down-regulating MAPK and PKA [135].

## Targeting cAMP–PKA pathway for cancer therapy

Given the involvement of cAMP–PKA pathway in tumor progression, targeting this pathway may be a potential strategy to treat cancer. The cAMP analogue 8-Cl-cAMP, also known as tacladesine, can inhibit the growth of colon cancer, breast cancer, lung cancer, fibrosarcoma and leukemia in vitro and in vivo [136, 137]. Due to the different binding affinity with PKA R subunits, 8-Cl-cAMP can down-regulate RIα and induce RIIβ expression [136]. Some researchers have proposed that 8-Cl-cAMP may also exert PKA-independent anti-tumor effects through its metabolite 8-Cl-adenosine and AKT2–PKBβ pathway [138, 139]. Although its anti-tumor mechanisms have not been fully elucidated, 8-Cl-cAMP has been tested in phase II clinical trials in patients with multiple myeloma and in phase I clinical trials in patients with metastatic colorectal cancer, but the results of these trials have not been disclosed (Table 2).

**Table 2:** Clinical study of antitumor drugs targeting cAMP–PKA pathway (from clinicaltrials.gov)

Identifier	Title	Cancer type	Locations
NCT00004902	Tocladesine in treating patients with recurrent or refractory multiple myeloma	Multiple myeloma and plasma cell tumor	Robert H. Lurie Comprehensive Cancer Center, Northwestern University Chicago, Illinois, United States
NCT00021268	Tocladesine in treating patients with recurrent or progressive metastatic colorectal cancer	Colorectal cancer	Jonsson Comprehensive Cancer Center, UCLA Los Angeles, California, United States
NCT00004864	Docetaxel and GEM 231 in treating patients with recurrent or refractory solid tumors	Unspecified adult solid tumor	Albert Einstein Comprehensive Cancer Center Bronx, New York, United States
NCT00004863	Paclitaxel and GEM 231 in treating patients with recurrent or refractory solid tumors	Unspecified adult solid tumor	Albert Einstein Comprehensive Cancer Center Bronx, New York, United States

Since cAMP has tumor suppressive roles in some types of cancer, increasing cAMP levels may inhibit these types of tumors. PDE inhibitors can suppress the depletion of cAMP. Notably, PDE inhibitors have been widely used in clinic to treat cardiovascular, respiratory and psychiatic diseases. The PDE3 inhibitors amrinone and milrinone are used as cardiotonic drugs. PDE4 inhibitor rolipram is a new anti-inflammatory drug for asthma treatment. PDE5 inhibitor sildenafil is a preferred drug for erectile dysfunction treatment [140]. In addition, some flavonoids natural products can also inhibit PDEs [141]. These PDE inhibitors may be repurposed to treat cancers in which cAMP has tumor-suppressive roles. In contrast, PDE activators may be developed to treat cancers that are promoted by cAMP signaling. Recently, a small-molecule compound that allosterically activates PDE4 long isoforms has been developed [142]. This prototypical PDE4 activator compound is able to reduce intracellular cAMP levels and inhibit cyst formation in the kidney [142]. It remains to know whether such kinds of PDE activators may be repurposed to treat some types of cancer.

Antisense oligonucleotide targeting the N-terminal PKA RIα is another choice to interfere with PKA signaling. PKA RIα knockdown leads to compensative expression of RIIβ and then inhibits tumor growth in a wide variety of tumor models [143]. The antisense oligonucleotide GEM231 has been evaluated in two phase I clinical trials (Table 2). The results of the phase I clinical trial of GEM231 in combination with docetaxel showed that the overall incidence of grade 3 adverse reactions, including fatigue, elevated aminotransferase, neutropenia and altered mental status, was 75% in 20 patients with refractory solid tumors during 39 cycles of treatment [144]. No subsequent clinical trial has been reported.

CREB may also be a target for cancer therapy. Currently, there is no specific CREB inhibitor. The small molecule XX-650-23 blocks the interaction between CREB and its co-activator CBP (CREB-binding protein) and then abrogates CREB-responsive gene expression, leading to AML cells apoptosis and cell-cycle arrest [145, 146]. Another feasible strategy is to downregulate CREB expression. Previous study demonstrates that GSKJ4, the histone lysine demethylases JMJD3/UTX inhibitor, can induce CREB degradation and inhibit AML cell growth [123]. Given that JMJD3/UTX have multiple targets, GSKJ4 may also inhibit cancer cell growth by targeting proteins other than CREB. Since CREB may be activated by CAMK in some types of cancer, inhibition of CAMK may be an alternative strategy to suppress CREB [147].

Although there are some PKA inhibitors that have tumor-suppressive effects in preclinical studies, no small-moleculae PKA inhibitors have been tested in clinical trials. It warrants further research to develop more small-molecule drugs targeting PKA and/or CREB, and evaluate their safety and efficacy in cancer therapy. In addition, repurposing some drugs that can act on PKA and CREB may be valuable to cancer therapy.

## Concluding remarks

cAMP signaling pathway is evolutionarily conserved and plays key roles in major physiological and pathological processes. The functions of cAMP signaling in tumors depend on cell type and specific environment. In many types of human tumors, cAMP–PKA–CREB pathway plays a tumor-promoting role. The abnormal activation of this pathway and its interaction with other signaling pathways are closely related to tumorigenesis, invasion, metastasis and drug resistance. In contrast, cAMP and CREB also have tumor-suppressive roles in some types of tumors, such as medulloblastoma and non-Hodgkin’s lymphoma [148–150]. While cAMP may inhibit tumor progression through downregulating cyclin A, Erk or other targets, PKA also reportedly inhibits some tumor-promoting signals. For example, PKA may inhibit the activity of mTORC1, an important tumor-promoter, through phosphorylating the mTORC1 component Raptor on Ser^791^ residue [4, 151]. It warrants further studies to determine whether targeting cAMP–PKA–CREB pathway is a feasible strategy to treat cancer and reverse cancer drug resistance.

## Data Availability

Not applicable.

## Abbreviations

ACs: Adenylyl cyclases
AKAPs: Protein kinase A anchoring proteins
ALL: Acute lymphocytic leukemia
AML: Acute myeloid leukemia
AR: Androgen receptor
ATF1: Activating transcription factor 1
CAMKK2: Calmodulin-dependent protein kinase kinase-2
cAMP: Cyclic adenosine monophosphate
CBP: CREB-binding protein
CIP4: CDC42 interacting protein 4
CLL: Chronic lymphocytic leukemia
CNG: Cyclic nucleotide gated ion channels
CRE: CAMP response element
CREB: CAMP response element binding protein
CREM: CRE modulator
EGFR: Epidermal growth factor receptor
Epac1/2: Exchange proteins directly activated by cAMP 1 and 2
FL-HCC: Fibrolamellar hepatocellular carcinoma
GBM: Glioblastoma
GEFs: Guanine nucleotide exchange factor
GLP-1: Glucagon-like peptide 1
GPCRs: G-protein-coupled receptors
HBV: Hepatitis B virus
HCC: Hepatocellular carcinoma
HCN: Hyperpolarization-activated cyclic nucleotide-gated channel
IGF-1R: Type-I insulin-like growth factor receptor
KID: Kinase inducible domain
NSCLC: Non-small cell lung cancer
PDEs: Phosphodiesterases
PDGFR: Platelet derived growth factor receptor
PGE2: Prostaglandin E2
PKA: CAMP-activated protein kinases
sAC: Soluble adenylyl cyclase
SCLC: Small cell lung cancer
VEGFR: Vascular endothelial growth factor receptor
